# Involving patients and carers in patient safety in primary care: A qualitative study of a co‐designed patient safety guide

**DOI:** 10.1111/hex.13673

**Published:** 2023-01-16

**Authors:** Rebecca L. Morris, Sally Giles, Stephen Campbell

**Affiliations:** ^1^ NIHR Greater Manchester Patient Safety Translational Research Centre, Centre for Primary Care and Health Services Research, Division of Population Health, Health Services Research and Primary Care University of Manchester Manchester UK

**Keywords:** co‐design, healthcare professional experience, improvement, patient experience, patient safety, primary care, UK

## Abstract

**Background:**

Involving patients is a key premise of national and international policies on patient safety, which requires understanding how patients or carers want to be involved and developing resources to support this. This paper examines patients' and carers' views of being involved in patient safety in primary care and their views of potentially using a co‐designed patient safety guide for primary care (PSG‐PC) to foster both involvement and their safety.

**Methods:**

A qualitative study using semistructured face‐to‐face interviews with 18 patients and/or carers in primary care. Interviews were transcribed and analysis was conducted using an inductive thematic approach.

**Results:**

Overall participants expressed enthusiasm for the PSG‐PC as a tool to support patients and carers to be involved in patient safety in primary care. However, for some participants being involved in patient safety was seen as taking on the role of General Practitioner and had the potential to add an additional workload for patients. Participants' willingness or ability to be involved in patient safety was influenced by a range of factors including an invisible, often underacknowledged role of everyday safety for patients' interactions with primary care; the levels of involvement that patients wanted in their care and safety and the work of embedding the PSG‐PC for patients into their routine interactions with primary care. Participants identified components of the PSG‐PC that would be useful to them, in particular, if they had a responsibility for caring for a family member if they had more complex care or long‐term conditions.

**Conclusion:**

Involving patients and carers in patient safety needs a tailored and personalized approach that enables patients and carers to use resources like the PSG‐PC routinely and helps challenge assumptions about their willingness and ability to be involved in patient safety. Doing so would raise awareness of opportunities to be involved in safety in line with personal preference.

**Patient or Public Contribution:**

Patient and public involvement were central to the research study. This included working in partnership to develop the PSG‐PC with patients and carers and throughout our study including in the design of the study, recruiting participants, interpretation of findings.

## 1 BACKGROUND

Patient safety is a key global health priority as health systems have increasing levels of demand for services alongside financial constraints, fragmentation, the changing role of technology in care and information transfer and an increase in long‐term condition management.^1^, ^2^, ^3^, ^4^, ^5^ Patient safety has been broadly defined as ‘the avoidance, prevention and amelioration of adverse outcomes or injuries stemming from the processes of healthcare’.^6^ For example, in primary care diagnostic and medication incidents, were most likely to result in harm or severe harm.^7^ There has been a focus on preventing the most common causes of harm such as prescribing, diagnosis and treatment in primary care.^3^, ^7^, ^8^, ^9^, ^10^ Patient–provider communication issues also contribute to patient safety incidents either directly or indirectly.^1^, ^11^ Yet who should be involved and responsible for patient safety remains unclear.^12^ Increasingly the role of patients and carers has been advocated as an additional component of patient safety to prevent serious incidents by preventing harm before it occurs to make care safer.^13^, ^14^, ^15^, ^16^, ^17^

Involving patients in their health care and research is an essential element for improving both the quality and safety of care and addressing inequalities within health and social care.^14^, ^18^ Involving patients in health care is one source of creating a resilient system that allows flexibility and adaptation within a complex healthcare system.^19^, ^20^, ^21^, ^22^, ^23^, ^24^ This flexibility may be what is needed to improve patient safety, and health outcomes and meet the needs of patient‐centred care.^20^, ^25^, ^26^ Patient safety issues can be identified throughout patient experiences with health care, from access or diagnosis to medication management, treatment and self‐management with primary care managing repeated uncertainties along episodes of care.^7^, ^27^, ^28^, ^29^ Involving patients more explicitly in their care needs to be considered within an understanding of the dynamic, nuanced nature of involvement as well as patient–clinician dynamics situated within the wider contextual factors which may influence involvement.^6^, ^15^, ^30^, ^31^ This approach builds on the growing debate within the wider patient safety literature. More broadly within the patient safety literature, there has been a shift from a ‘Safety‐I’ perspective (which can be defined as a context in which as few things go wrong as possible) to a ‘Safety‐II’ approach (which focuses on how things go well in everyday work by understanding the uncertainties and trade‐offs).^32^ It has been argued that these two perspectives on safety reflect two distinct but complimentary views of patient safety.^33^ There is a need to learn from everyday work in the development of initiatives for patient safety that build on a deeper understanding of safety.^33^

Assumptions are often made within the policy and broader literature that patient involvement is an equally desired role by all patients without recognizing the role of the system, contextual or individual (e.g., health literacy) factors that might influence an individual's capacity or desire to be involved.^30^ How patients identify their eligibility for health care is influenced by multiple factors, from the individual themselves, and their social contexts through to macro‐level influences on services from resource allocation to service design, this complex interplay of factors have been termed candidacy.^34^ This is important when considering that patients and their carers have a unique perspective as they move within and across system transitions and have the potential to reduce variability experienced from health care which may have an impact on outcomes.^20^, ^35^ The majority of research on patient safety in primary care has been descriptive, with few studies focusing on interventions aimed at involving patients in their safety.^1^, ^27^, ^36^, ^37^, ^38^, ^39^ Patients' capacity or willingness to raise safety concerns may be influenced by previous experiences of health care, especially where previous experiences have left them feeling vulnerable.^8^, ^12^, ^36^, ^40^, ^41^, ^42^ For example, patients living with multiple long‐term conditions or with more complex care needs experience duplication and fragmentation of care and experience.^36^, ^43^

One approach to involving patients in patient safety is to create an ongoing dialogue which builds trust, clarifies expectations and ensures understanding between patients and healthcare professionals.^10^, ^39^, ^44^ Yet this approach is based on an assumption that everyone will want and be able to be actively involved in their care and patient safety. There has been limited research which has examined what this would look like in practice and whether patients have the willingness or capacity to take on this additional work.^38^, ^45^, ^46^ This expectation of the role of patients in patient safety needs to be examined to ensure that initiatives to involve patients do not create or reinforce inequity or compound patient safety risks.^47^

There have been tools and handbooks developed for patients to support their involvement in their care and safety in secondary care (including safety tips, treatment plans and fall prevention) but an equivalent version does not exist in primary care.^15^, ^48^, ^49^, ^50^, ^51^, ^52^, ^53^ This study is part of a wider project to develop and test the patient safety guide for primary care (PSG‐PC).^15^, ^36^ The PSG‐PC supports patients and carers to address key patient safety questions and identify key points where they can make their primary care interactions safer and be active partners in their care. The PSG‐PC has been developed using an experience‐based co‐design approach in partnership with patients, carers, members of the public and healthcare professionals (including GPs and pharmacists) and the development has been reported in detail elsewhere.^15^, ^36^, ^47^, ^54^, ^55^ The PSG‐PC was co‐designed to support effective communication between patients and clinicians, which was considered a key component of involving patients and carers in patient safety in primary care.^15^ The aim of this study was to explore patients' and carers' views of being involved in patient safety in primary care and their views of potentially using the PSG‐PC to support involvement.

## 2 METHOD

### 2.1 Design

**Figure 1:**
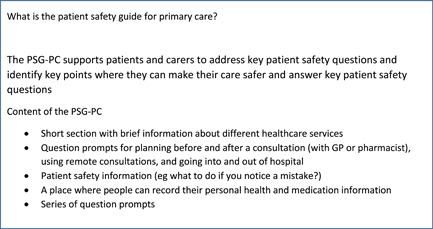
The PSG‐PC components. PSG‐PC, patient safety guide for primary care.

An in‐depth qualitative study design was adopted using semistructured interviews which examined patients' and carers' views of being involved in patient safety in primary care and their views of potentially using a PSG‐PC (see Figure 1) to support involvement. Semistructured interviews were chosen to allow participants to discuss without limitation the areas they recognized as most important as well as to include topics that the research team, including members of the patient and public involvement group for the study, identified as relevant.^56^ the PSG‐PS has been developed using a participatory approach based on the experience‐based co‐design approach,^15^ for this part of the wider project we aimed to explore the views of people who had not been involved in the development of the prototype to enhance the potential acceptability and feasibility of the intervention.^57^ This study is part of a wider project to develop and test the PSG‐PC,^15^, ^36^ and used a prototype of the PSG‐PC to explore patient and carers' views of being involved in patient safety and using a tool such as the PSG‐PC to support this.

### 2.2 Recruitment

Patients and carers were recruited using a snowball sampling approach through advertisements and contacts including with voluntary groups and community centres across Greater Manchester and social media (Twitter).^58^ Eighteen patients and/or carers were interviewed. Fourteen participants were female, and 18 were White. We recruited participants from a range of characteristics of different socioeconomic status, geographical areas, gender, experiences of using services and different numbers and types of conditions. Participants ranged from having no long‐term conditions to a maximum of seven long‐term conditions (see Table 1). As this study was examining a prototype that was only available in English, potential participants were excluded if they could not speak or read English. They were invited to contact the research team by email or phone to express interest, and potential participants were sent a participant information sheet. Recruitment was completed when no new themes that were relevant to the aims of the project emerged from the data.^59^

**Table 1:** Participant demographics

Participant ID	Age	Gender	Employment status	Health condition
PS01	72	Female	Retired	Chronic obstructive pulmonary disease (COPD) or asthma
PS02	69	Female	Retired	Diabetes; arthritis
PS03	65	Male	Kitchen porter	Thyroid problem
PS04	71	Female	Retired	Chronic obstructive pulmonary disease (COPD) or asthma; eczema
PS05	45	Male	Town and country planning	Chronic fatigue syndrome; anxiety or depression
PS06	53	Female	Carer	Arthritis; anxiety or depression; lupus
PS07	58	Male	Retired	Angina or heart attack; high blood pressure; kidney problems; COPD or asthma; stomach problems; arthritis; anxiety or depression
PS08	58	Female	Administrator	None reported
PS09	25	Female	Postgraduate student and project manager intern	Polycystic ovary syndrome
PS10	58	Female	Medically retired civil servant	Thyroid problem
PS11	69	Female	Retired	High blood pressure; arthritis
PS12	71	Male	Retired	High blood pressure; anxiety or depression; high cholesterol; heart murmur
PS13	71	Female	Retired	Stomach problems; arthritis; anxiety or depression
PS14	52	Female	Teaching assistant	None reported
PS15	64	Female	Retired	High cholesterol
PS16	40	Female	Project work	None reported
PS17	34	Female	Volunteer at primary school	Anxiety or depression; schizoaffective disorder
PS18	34	Female	Student	COPD or asthma; stomach pains; anxiety or depression

### 2.3 Data collection

Interviews were conducted by either R. L. M. or S. G. face‐to‐face between January and March 2017. Written consent was obtained from all participants. The topic guide was developed by researchers in collaboration with the patient and public involvement contributors. Interviews were conducted in participants' homes. Interviews ranged from 22 to 59 min (average of 39 min). Interview participants were offered a £20 voucher to compensate them for their time. Reimbursing participants for participation is becoming more commonplace which may influence peoples' willingness to participate in research, however, it has been argued that is unethical to not reimburse participants to in part address power imbalances between paid researchers and unpaid participants.^60^, ^61^ To reduce the potential of the payment influencing the participants' comments, it was explained to participants that the research was interested in their opinions and that there were no right or wrong answers and it was explained that the vouchers were reimbursement for their time and were free not to answer questions and could end the interview at any time.

The interviews were digitally recorded and professionally transcribed. Interviews explored openly patients' and carers' views of being involved in patient safety in primary care and their views of potentially using the PSG‐PC to support involvement, and what they liked and did not like about the PSG‐PC. Interviews took an open approach, using vignettes to explore the limitations of the guide and how people could use it in practice which enabled participants to examine in depth what would or would not be acceptable for them in line with a person‐based approach to intervention development.^57^ This included exploring their experiences of using primary care services, health and medication management, giving feedback, their view on the PSG‐PC and what information is important, or not, to support people to be involved in patient safety. The topic guide was modified iteratively throughout data collection and analysis.

### 2.4 Data analysis

Data analysis was ongoing throughout the study and followed an inductive thematic approach.^62^, ^63^ All authors met regularly to discuss emerging themes. All authors analysed transcripts and commented on the interpretation of the data and agreed on the key themes and concepts. Key themes were identified through discussion. Key themes and quotes were circulated to all the authors for comments and discussion. A final set of themes and subthemes were agreed upon by all authors. Nvivo 11 qualitative analysis software was used to support analysis. The sample size was determined by thematic saturation, which was identified when there was a point where no new themes or codes were developed from the analysis.^64^

### 2.5 Patient and public involvement

A patient and public involvement group was established to support the wider study to co‐develop the PSG‐PC along with other stakeholders and information about that has been detailed elsewhere.^15^ Our patient and public involvement group were involved in the qualitative study to help design and format the questions and support recruitment. R. L. M. also presented the initial themes and led a discussion about what they meant in terms of developing the PSG‐PC and interpreting types of involvement in patient safety that participants expressed which fed into the subsequent refinement of the PSG‐PC.^15^, ^36^

## 3 RESULTS

Eighteen patients and/or carers were interviewed. Fourteen participants were female, and 18 were White. Eight participants were employed, eight were retired and two were students. Participants' ages ranged from 34 to 72 years old (mean age of 56 years) (see Table 1). The main themes related to the invisible role of everyday safety for patients with interactions with primary care; the levels of involvement of patients in patient safety in primary care and the role of the PSG‐PC and the work of embedding the PSG‐PC for patients into routine interactions with primary care.

### 3.1 The invisible role of everyday safety for patients with interactions with primary care

Participants described initially their contact with primary care and their role in patient safety in an implicit way. Participants' accounts detailed everyday experiences where they identified issues that could be considered potential safety issues. The PSG‐PC could help prevent this as it includes a prompt to consider medication allergies to make explicit their role in identifying medications that they have had previous allergic reactions to as patients moved across healthcare settings. For example, one participant was prescribed multiple times a selective serotonin reuptake inhibitor which they were allergic to and was recorded in their patient records and they described a breakdown in trust as they did not feel listened to or in partnership in their own care. The importance of systems and tools at home to manage complex medications to prevent safety issues was often unrecognized but when they broke down it highlighted the importance of them generally preventing medication‐related patient safety issues. One participant described the importance of systems to manage their medications to avoid accidental overdoses of medication to manage schizoaffective disorder:

I wouldn't survive without my pillbox … I do my pills every week. I think if I didn't have that I'd probably be in a bit of a state. One time I accidentally took an overdose of the sodium valproate … I rang the support team and they said, well, you need to get to hospital straight away. (PS17)

Most participants described supporting family members (usually elderly relatives) or friends as informal carers and described it as often being unrecognized. They detailed the complexity of supporting relatives and coordinating care whilst supporting their family members. This often involved remembering the person they were caring for (often complex) medical history, multiple appointments as well as the everyday logistics of getting them to appointments. Participants described adopting this role with little support from health services and the additional burden to them. The following participant described the challenges of caring for their mother and getting her to attend hospital appointments. Her mother had multiple health conditions, needed a wheelchair and had memory problems and by the time they arrived at the consultation, she had often forgotten key things that needed to be discussed.

I think it [PSG‐PC] would [useful]. Because [mother] was very reluctant to go to a consultant at the hospital. Mainly because of the disruption of going … So, if you've got what you want to ask written down, you can concentrate on making her comfortable in our case, and you could always refer to this when you got there. You're not worrying about oh will I remember everything. (PS02)

### 3.2 Levels of involvement of patients in patient safety in primary care and the role of the PSG‐PC

Participants' accounts detailed a continuum of levels of involvement in patient safety in primary care that they would feel willing or able to adopt which would influence whether they would want to use the PSG‐PC. For some participants, this meant that they would not use the PSG‐PC, other participants described selectively choosing sections that were relevant to them and not using other sections. This continuum was not necessarily static but depended on a combination of contributing factors (such as their health status, their perception of the role of the patient or implicit boundaries of responsibility for different elements of care). For some participants, they viewed being involved in patient safety as part of being a ‘patient’ and their patient identity and working in partnership with their GP.

I think that is good for people to have ownership over their medical problems anyway and work, sort of, almost in partnership with the GP to figure out, you know, how to move forwards. (PS09)

Conversely, some participants described their involvement with their healthcare professional in more transactional terms of trying to get the most out of a short consultation which threatens the development of trust and reinforces power dynamics that prioritize the professionals' agenda with limited to develop relationships. For example, the following participant described the challenges of a 10‐min appointment and felt pressure to not overrun whilst also trying to raise issues that they wanted to discuss:

I think [consultation] could be a bit longer … it's about ten minutes. So, you just have to keep to the point. It's not social … It's more to the point and that's it. (PS02)

A few participants did not feel they had an explicit role in patient safety as they felt that this was within the healthcare professionals' roles to maintain the patient records as accurate and up to date. For these participants, initially, their accounts depicted a clear boundary of responsibility in healthcare settings with them not necessarily identifying their role as part of an implied ‘safety net’ but the PSG‐PC challenged their assumptions about their role and they could identify the potential to ensure information held about them is accurate.

And the doctors, with all the computers and what have you should know what I'm on anyway. And they should be able to refer back to that [patient record] … not always true … I think it [PSG‐PC] would be a very good idea. (PS02)

One participant recognized that the questions and prompts in the PSG‐PC may have helped them to plan as they became ill and may have helped to avoid delays in diagnosis or management which they experienced.

This [to feedback] never occurred to me when I first became poorly and I think I could have prevented a lot of what went wrong … You know, my management of my illness a lot better. (PS17)

Another component of involving patients in patient safety can include being willing to feedback (either positive or negative) to healthcare professionals. Some participants were comfortable giving and described it as just part of being an informed patient.

Yeah, it's good for them to know, good for the patient to know, you know, feedback what's happening, what's going to happen? You know, say how you feel, and stuff like that, you know. (PS03)

Those participants who had provided feedback described their uncertainty about how to provide feedback in general practice and often felt unsupported. For example, the following participant did not know how to make a complaint about their GP and felt disempowered when told to speak to the GP directly:

I've made a formal complaint in the past about one of the GPs, and I did a letter to the practice manager because that's the only thing I knew … I didn't know anything about the complaints procedure, all I knew is to write to the practice manager rather than the GP themselves, but then they asked me in to speak to the actual GP themselves, so I didn't take it any further. (PS16)

### 3.3 The work of embedding the PSG‐PC for patients into routine interactions with primary care

Participants described a range of ways that they could identify the PSG‐PC that may be relevant to themselves or others. For example, most participants identified that the PSG‐PC could be used as an aide memoire to support them during a consultation to remember key points or questions they wanted to raise. Other participants reflected that the PSG‐PC layout was logical linking with the sequencing of a consultation and could help reduce the ‘door knob’ effect which is particularly important for participants managing long‐term or complex conditions as they carried information across healthcare providers. The following participant described how they would get anxious before an appointment and then forget the main points they wanted to discuss with their GP:

Yeah, you could have that as … a prompt sort of before you go in and say, right … Whereas at the minute I think you're so worked up at going anyway and, like you say, you do forget, you know, when you come out of it and you think, oh, maybe I should have asked that, but then that time's gone … so, it's making the most of that time. (PS01)

Some participants thought the PSG‐PC would be particularly useful as a prompt if trying to introduce sensitive or potentially embarrassing symptoms.

[PSG‐PC] just gives you that route as to train of thought … you know, phrasing wise … How can I announce it, do I just give it to the GP blunt … a prompt, you know memory wise, you know, how to introduce it … sometimes it's difficult to think of ways to, sort of, like, introduce the, you know, whatever you've got. (PS03)

However, in contrast for a few participants having a prompt could potentially be an additional burden or distraction from a consultation and additional task loading could overburden them. One participant described their experience of consultations where they felt that adding in the guide would distract them as they already found it challenging to interpret the medical terms.

And sometimes when I'm in a session with the doctor, I'm so absorbed by trying to get out what the issue is, and trying to talk to them, even though I have notes of, okay, have I talked about this, and this, I can't comprehend them, it's like it becomes all jargon when I'm inside the appointment. (PS18)

Whilst most participants recognized that the PSG‐PC could be useful it was typically for particular, more relevant sections and they would selectively use components depending on what they needed at that time.

Just from what I've seen maybe there were things there that weren't important for me but I know that they are important for other people who perhaps have less understanding or through age are not comfortable. (PS13)

Participants were shown a paper version of the guide and participants discussed a range of opinions for using an online or mobile application‐based version with the majority wanting both options as a way of making it accessible and inclusive.

Both [paper and digital formats] for me … I think I prefer paper, but obviously, I know the way the world is going most things are going electronically anyway … I think that some people feel that they are being, not left out, sidelined because they haven't got the accessibility to a computer, whereas everyone can get a pen or pencil. (PS08)

The majority of participants expressed that they would use the PSG‐PC, however, one participant felt that using the PSG‐PC would involve them taking on the role of the GP.

I actually think this booklet, it's like the patient has to do the GP's job for him. (PS16)

## 4 DISCUSSION

The study analysis highlighted a more nuanced understanding of how patients and carers want or the extent to which they feel able to be involved in patient safety in primary care. In particular, it highlighted the invisible work and often implicit work that patients and carers are already doing to foster fostering patient safety (e.g., avoiding serious adverse events by identifying medications for which participants had a known allergy). The PSG‐PC could support patients and carers to draw out this tacit patient safety knowledge and make it explicit and therefore has the potential to support patients to be active in patient safety and their care. However, for some participants, the PSG‐PC could add an additional burden when they are already overwhelmed with their health or the responsibility of caring for a family member. Other studies have highlighted patients who may wish to raise safety concerns may be vulnerable to or perceive a power imbalance that may influence their willingness or ability to raise patient safety issues if they would like to.^12^, ^40^ The systems in which healthcare provision occurs can reinforce existing inequities and influence patients' ability to raise problems or their perception of entitlement to care which creates a context in which involvement in patient safety may be limited.^65^ Despite this, the majority of participants in this study considered the PSG‐PC that could support their involvement in patient safety, and they would selectively use the parts of the guide which were most relevant to their particular context. This suggests that the PSG‐PC might be considered a complex intervention with the safety issues that it aims to address are dynamic and emergent. This is an important component of conceptualizing how these types of interventions aimed at involving patients in patient safety can be responsive to support the adoption or nonadoption of an intervention in the future.^66^ Embedding the PSG‐PC more broadly into care and consultation pathways was identified as an important part of implementing the PSG‐PC by patients but was deemed limited unless healthcare professionals were also engaged with its use.

Medication management is one area where patients and carers often more explicitly identify patient safety issues.^67^ Participant accounts often depicted taking this role for granted and not linking it to patient safety which may influence their willingness to adopt an explicit role in patient safety as this could be seen as additional work rather than recognizing their existing contribution to enhancing safety. Furthermore, whilst patients may have a more clearly identifiable role in medication management for patient safety, the role of communication is also an important contributing factor.^15^, ^40^ This is a multifactorial component with the timeliness and clarity of information a key dimension of fostering an environment in which patient involvement in patient safety may occur.^12^, ^15^, ^40^ Participants' accounts depicted assumptions about the information that the general practitioner had available during the consultation and the accuracy of the patient record. It was often taken for granted by participants that this information would be up‐to‐date, accurate and used to inform consultations and for participants that had this belief, it limited the information participants felt was necessary to include during a consultation. However, patient records may be poorly maintained, have missing information and have inconsistencies between entries.^68^ This is particularly important as the information that patients or carers disclose during a consultation may be based on a potentially erroneous assumption that may have consequences for patient safety (e.g., a medication with known allergies) and places the burden of responsibility on patients to monitor medication prescriptions. It is important to understand these uncertainties and trade‐offs conceptually from a Safety‐II perspective.^33^ The PSG‐PC was identified as being able to support this as it may foster better communication and transparency so both patients and healthcare professionals have a more shared understanding of what has happened and what may need to be done in the future.

Another area of patient involvement in patient safety which has received the most attention is the role of patients providing feedback to healthcare providers and particularly in hospital settings.^30^, ^69^ For example, in the UK national patient safety syllabus a key component is learning from events and lived experiences but this is based on an assumption that people know how and where to give feedback.^14^, ^15^, ^70^ Participants' accounts within this study challenged this assumption as participants' accounts depicted a range of preferences and experiences for giving feedback in primary care with most not knowing how to give feedback or having negative experiences if they had given feedback.

Previous research has examined how patients conceptualize responsibility for health care and the boundaries between patient and professional roles that involving patients in patient safety may occupy.^12^ This study extends this to identify how the role that a patient or carer may identify as being willing or able to adopt to foster patient safety may influence their use of the PSG‐PC. This is of particular importance as much of the national and international policy and academic literature highlights the importance of involving patients in patient safety but there is considerably less focus on how to do it.^1^, ^2^, ^3^, ^4^, ^5^, ^6^, ^7^, ^8^, ^9^, ^10^, ^11^, ^12^, ^14^ Accounts depicted a continuum of levels of involvement in patient safety as opposed to a categorical approach (i.e., they are involved or not) which was flexible and dynamic depending on a range of contributory factors. Similarly, Heavey et al.^12^ found with patients recently discharged from the hospital, that their narratives of responsibility for safety in the hospital were based on either personal expertise and/or a duty of self‐care or alternatively an expectation of safety was the focus of professionals duty and expertise. Accounts in this study identified a varying role of expectation for involvement with accounts ranging from more individualized, personal responsibility, which resonated patient safety conceptually with self‐care, through to it being the GPs role. This influenced the extent to which participants felt able to shift the boundaries and expectations of their role which in turn influenced their willingness or ability to share responsibility for patient safety and the PSG‐PC.^71^, ^72^

### 4.1 Strengths and limitations

This study draws on patient and carers' experiences and prospective accounts using the PSG‐PC to support them to be more active in patient safety which extends previous work which identifies that patients should be involved but crucially not *how* to involve them using a co‐design approach.^20^ This is a strength of this study as it supports an understanding of how different participants identify if they feel able and willing to be involved in patient safety in primary care. One limitation of this approach is that, while we obtained participants' initial thoughts participants had not used the PSG‐PC in practice. Future work needs to examine how patients and carers use the PSG‐PC and whether it is acceptable.^28^ In this study, due to resource constraints, only patients and carers were interviewed and it is a limitation that we did not interview other stakeholders. Interviews were conducted by members of the research team and whilst participants were asked open questions and purposefully questioned about what they did not like about the guide, in line with intervention development studies.^57^ Participants were recruited through a snowball sampling approach and whilst the team recruited people from a range of backgrounds there is a limitation that only the views of people who volunteer are reported and the views of people who may not have the time or capacity were not included.^73^ Participants' views were based on a paper version of the PSG‐PC and future research should explore the development of mobile phone applications. One main limitation of the study is that the PSG‐PC was only available in English as this was a prototype and the first stage in developing and testing the PSG‐PC, further work is needed to culturally adapt the PSG‐PC or develop versions for marginalized groups that may have specific needs (e.g., text‐to‐speech option for people with vision impairment). The changing context of health care since the start of the severe acute respiratory syndrome coronavirus 2 (SARS‐CoV‐2) COVID‐19 pandemic has acutely brought to the fore the need for a nuanced approach to patient involvement in patient safety that is responsive to changing models of care (e.g., video chat).

## 5 CONCLUSION

By examining patients' and carers' views of being involved in patient safety this study shows the need for a nuanced and personalized approach. The PSG‐PC has the potential to support patients' and carers' involvement. This study identified challenges to assumptions about patients' and carers' willingness and ability to be involved in patient safety (e.g., that all patients want to be involved in patient safety) and the potential adoption of the PSG‐PC in routine practice.

## AUTHOR CONTRIBUTIONS

Rebecca L. Morris led the design of project, data collection, analysis, interpretation and drafted the manuscript. Stephen Campbell conceived the project and Sally Giles collected data. All authors developed the analysis and interpretation and critically contributed to the prototype development and manuscript.

## CONFLICT OF INTEREST

The authors declare no conflict of interest.

## ETHICS STATEMENT

Ethical approval for this study was granted by Yorkshire & The Humber‐Sheffield Research Ethics Committee (REC reference: 16/YH/0496).

## Supporting information

Supporting information.

## Data Availability

Research data are not shared.

## References

[1] Panesar SS , de Silva D , Carson‐Stevens A , et al. How safe is primary care? A systematic review. BMJ Qual Saf. 2015;25(7):544‐553. 10.1136/bmjqs-2015-004178 26715764

[2] Sheikh A , Panesar SS , Larizgoitia I , Bates DW , Donaldson LJ . Safer primary care for all—a global imperative. Lancet Global Health. 2013;1(4):e182‐e183. 25104342 10.1016/S2214-109X(13)70030-5

[3] Health Education England . National Patient Safety syllabus 2.0. 2022. Accessed June 15, 2021. https://www.hee.nhs.uk/our-work/patient-safety

[4] WHO . Delivery Quality Health Services: A Global Imperative for Universal Health Coverage. World Health Organization; 2016.

[5] WHO . Towards eliminating avoidable harm in health care. Third Draft. 2021. Accessed September 20, 2021. https://cdn.who.int/media/docs/default-source/patient-safety/gpsap/global-patient-safety-action-plan-2021-2030_third-draft_january-2021_web.pdf?sfvrsn=6767dc05_15&download=true

[6] Vincent C . Patient Safety. Wiley‐Blackwell; 2010.

[7] Panagioti M , Khan K , Keers RN , et al. Prevalence, severity, and nature of preventable patient harm across medical care settings: systematic review and meta‐analysis. BMJ. 2019;366:l4185. 31315828 10.1136/bmj.l4185 PMC6939648

[8] Allen D , Braithwaite J , Sandall J , Waring J . Towards a sociology of healthcare safety and quality. Sociol Health Illn. 2015;38(2):181‐197. 26679563 10.1111/1467-9566.12390

[9] NHS England . Primary care services. 2016. Accessed August 1, 2020. https://www.england.nhs.uk/participation/get-involved/how/primarycare/

[10] Daker‐White G , Hays R , McSharry J , et al. Blame the patient, blame the doctor or blame the system? A meta‐synthesis of qualitative studies of patient safety in primary care. PLoS One. 2016;10:8. 10.1371/journal.pone.0128329 PMC4526558 26244494

[11] Ricci‐Cabello I , Marsden KS , Avery AJ , et al. Patients evaluations of patient safety in English general practices: a cross‐sectional study. Br J Gen Pract. 2017;67(660):e474‐e482. https://pubmed.ncbi.nlm.nih.gov/28583945/ 28583945 10.3399/bjgp17X691085 PMC5565856

[12] Heavey E , Waring J , De Brún A , Dawson P , Scott J . Patients' conceptualizations of responsibility for healthcare: a typology for understanding differing attributions in the context of patient safety. J Health Soc Behav. 2019;60(2):188‐203. 31113253 10.1177/0022146519849027

[13] Morris RL , Stocks SJ , Alam R , et al. Identifying primary care patient safety research priorities in the UK: a James Lind Alliance Priority Setting Partnership. BMJ Open. 2018;8:e020870. 10.1136/bmjopen-2017-020870 PMC5855454 29490970

[14] NHS England and NHS Improvement . The NHS Patient Safety Strategy: safer culture, safer systems, safer patients. 2019. Accessed December 2, 2019. https://improvement.nhs.uk/documents/5472/190708_Patient_Safety_Strategy_for_website_v4.pdf

[15] Morris RL , Ruddock A , Gallacher K , Rolfe C , Giles S , Campbell S . Developing a patient safety guide for primary care: a co‐design approach involving patients, carers and clinicians. Health Expect. 2021;24(1):42‐52. 33142022 10.1111/hex.13143 PMC7879544

[16] NHS England . Framework for involving patients in patient safety. 2021. Accessed September 20, 2021. https://www.england.nhs.uk/wp-content/uploads/2021/06/B0435-framework-for-involving-patients-in-patient-safety.pdf

[17] NHS England . Patient and public participation in commissioning health and care: statutory guidance for clinical commissioning groups and NHS England. 2017. Accessed September 20, 2021. https://www.england.nhs.uk/wp-content/uploads/2017/05/patient-and-public-participation-guidance.pdf

[18] Vincent CA . Patient safety: what about the patient? Qual Saf Health Care. 2002;11:76‐80. 12078376 10.1136/qhc.11.1.76 PMC1743559

[19] Healthwatch . Equalities diversity and inclusion plan 2021‐22. 2021. Accessed September 20, 2021. https://www.healthwatch.co.uk/sites/healthwatch.co.uk/files/20210616%20Equalities%20and%20Diversities%20Action%20Plan%20COMMS%20EDIT.pdf

[20] O′Hara JK , Aase K , Waring J . Scaffolding our systems? Patients and families ‘reaching in’ as a source of healthcare resilience. BMJ Qual Saf. 2019;28:3‐6. 10.1136/bmjqs-2018-008216 29764929

[21] Kannampallil TG , Schauer GF , Cohen T , Patel VL . Considering complexity in healthcare systems. J Biomed Inf. 2011;44:943‐947. 10.1016/j.jbi.2011.06.006 21763459

[22] Braithwaite J , Wears RL , Hollnagel E . Resilient health care: turning patient safety on its head. Int J Qual Health Care. 2015;27:418‐420. 26294709 10.1093/intqhc/mzv063

[23] Wears RL , Hollnagel E , Braithwaite J . Resilient Health Care Volume 2: The Resilience of Everyday Clinical Work. Ashgate Publishing Limited; 2013.

[24] Hollnagel E , Braithwaite J , Wears RL , et al. Resilient Health Care. Ashgate Publishing Limited; 2013.

[25] Aase K , Waring J , Schibevaag L . Researching Quality in Care Transitions: International Perspectives. Palgrave Macmillan; 2017.

[26] Waring J , Marshall F , Bishop S , et al. An ethnographic study of knowledge sharing across the boundaries between care processes, services and organisations: the contributions to ‘safe’ hospital discharge. Health Serv Deliv Res. 2014;2:1‐160. 25642570

[27] Vincent C , Amalberti R . Safer Healthcare: Strategies for the Real World. Springer; 2016. 29465922

[28] Jerak‐Zuiderent S . Certain uncertainties: modes of patient safety in healthcare. Soc Stud Sci. 2012;42:732‐752. 23189612 10.1177/0306312712448122

[29] Balogh E , Miller BT , Ball JR . Improving Diagnosis in Health Care. Institute of Medicine; 2015. Accessed December 10, 2019. http://www.nationalacademies.org/hmd/Reports/2015/Improving-Diagnosis-in-Healthcare.aspx 26803862

[30] Murray J , Hardicre N , Birks Y , O′Hara J , Lawton R . How older people enact care involvement during transition from hospital to home: a systematic review and model. Health Expect. 2019;22:883‐893. 31301114 10.1111/hex.12930 PMC6803411

[31] Bastiaens H , Van Royen P , Pavlic DR , Raposo V , Baker R . Older people's preferences for involvement in their own care: a qualitative study in primary health care in 11 European countries. Patient Educ Couns. 2007;68:33‐42. 17544239 10.1016/j.pec.2007.03.025

[32] WHO. From Safety‐I to Safety‐II: A White Paper. WHO; 2015. Accessed May 2, 2022. https://www.england.nhs.uk/signuptosafety/wp-content/uploads/sites/16/2015/10/safety-1-safety-2-whte-papr.pdf

[33] Verhagen MJ , de Vos MS , Sujan M , Hamming JF . The problem with making Safety‐II work in healthcare. BMJ Qual Saf. 2022;31:402‐408. 10.1136/bmjqs-2021-014396 35304422

[34] Dixon‐Woods M , Cavers D , Agarwal S , et al. Conducting a critical interpretive synthesis of the literature on access to healthcare by vulnerable groups. BMC Med Res Methodol. 2006;6:35. 16872487 10.1186/1471-2288-6-35 PMC1559637

[35] Hollnagel E . FRAM: The Functional Resonance Analysis Method. CRC Press; 2012.

[36] Morris RL , Gallacher K , Hann M , et al. Protocol for a non‐randomised feasibility study evaluating a codesigned patient safety guide in primary care. BMJ Open. 2021;11:e039752. 10.1136/bmjopen-2020-039752 PMC7818830 33472773

[37] De Wet C . An Overview of patient safety in primary care. 2012. Accessed December 2, 2019. https://www.nes.scot.nhs.uk/media/2075343/an-overview-of-patient-safety-in-primary-care-nov-12.pdf

[38] Scott J , Dawson P , Jones D . Do older patients' perceptions of safety highlight barriers that could make their care safer during organisational care transfers? BMJ Qual Saf. 2012;21:112‐117. 10.1136/bmjqs-2011-000300 22069114

[39] Rowley E , Wright N , Waring J , Gregoriou K , Chopra A . Protocol for an exploration of knowledge sharing for improved discharge from a mental health ward. BMJ Open. 2014;4:e005176. 10.1136/bmjopen-2014-005176 PMC4185338 25273812

[40] Sutton E , Eborall H , Martin G . Patient involvement in patient safety: current experiences, insights from the wider literature, promising opportunities? Public Manag Rev. 2015;17(1):72‐89.

[41] Davis RE , Jacklin R , Sevdalis N , Vincent CA . Patient involvement in patient safety: what factors influence patient participation and engagement? Health Expect. 2007;10:259‐267. 17678514 10.1111/j.1369-7625.2007.00450.x PMC5060404

[42] Davis RE , Sevdalis N , Vincent CA . Patient involvement in patient safety: how willing are patients to participate? BMJ Qual Saf. 2011;20:108‐114. 10.1136/bmjqs.2010.041871 21228083

[43] Stafford M , Steventon A , Thorlby R , Fisher R , Turton C , Deeny S . Briefing: Understanding the Health Care Needs of People with Multiple Health Conditions. The Health Foundation; 2018.

[44] Reason J . Human error: models and management. BMJ. 2000;320:768‐770. 10720363 10.1136/bmj.320.7237.768 PMC1117770

[45] Shippee ND , Shah ND , May CR , Mair FS , Montori VM . Cumulative complexity: a functional, patient‐centered model of patient complexity can improve research and practice. J Clin Epidemiol. 2012;65:1041‐1051. 22910536 10.1016/j.jclinepi.2012.05.005

[46] Ward V , Smith S , House A , Hamer S . Exploring knowledge exchange: a useful framework for practice and policy. Soc Sci Med. 2012;74:297‐304. 22014420 10.1016/j.socscimed.2011.09.021

[47] Stocks SJ , Donnelly A , Esmail A , et al. Frequency and nature of potentially harmful preventable problems in primary care form the patient's perspective with clinician review: a population‐level survey in Great Britain. BMJ Open. 2018;8(6):e020952. 10.1136/bmjopen-2017-020952 PMC6009615 29899057

[48] John Hopkins Medicine . The John Hopkins Hospital patient and family handbook. 2019. Accessed December 10, 2019. https://www.hopkinsmedicine.org/the_johns_hopkins_hospital/_docs/the-johns-hopkins-hospital-patient-handbook.pdf

[49] NHS Imperial College Health Care . During your stay. 2017. Accessed September 20, 2021. https://www.imperial.nhs.uk/patients-and-visitors/patient-information/your-stay-in-hospital/during-your-stay

[50] NHS Imperial College Health Care . Where to go for care. 2022. Accessed September 20, 2021. https://www.imperial.nhs.uk/patients-and-visitors/patient-information/where-to-go-for-care

[51] Danish Society for Patient Safety . Patient Handbook: a patient's guide to safer hospital stay. 2016. Accessed December 10, 2019. http://arkiv.patientsikkerhed.dk/media/655535/patient_handbook2_.pdf

[52] Wright J , Lawton R , O′Hara J , et al. Improving patient safety through the involvement of patients: development and evaluation of novel interventions to engage patients in preventing patient safety incidents and protecting them against unintended harm. NIHR Journals Library (Southampton). 2016. 27763744

[53] Hjelmfors L , Strömberg A , Friedrichsen M , Sandgren A , Mårtensson J , Jaarsma T . Using co‐design to develop an intervention to improve communication about the heart failure trajectory and end‐of‐life care. BMC Palliat Care. 2018;17(1):85. 29890974 10.1186/s12904-018-0340-2 PMC5996457

[54] Bate P , Robert G . Experience‐based design: from redesigning the system around the patient to co‐designing services with the patient. Qual Saf Health Care. 2006;15(5):307‐310. 17074863 10.1136/qshc.2005.016527 PMC2565809

[55] The Health Foundation . Person‐centred care made simple. 2014. Accessed December 10, 2019. http://www.health.org.uk/sites/health/files/PersonCentredCareMadeSimple.pdf

[56] Taylor AK , Kingstone T , Briggs TA , et al. ‘Reluctant pioneer’: a qualitative study of doctors' experiences as patients with long COVID. Health Expect. 2021;24(3):833‐842. 33749957 10.1111/hex.13223 PMC8235894

[57] Yardley L , Ainsworth B , Arden‐Close E , Muller I . The person‐based approach to enhancing the acceptability and feasibility of interventions. Pilot Feasibility Stud. 2015;1:37. 27965815 10.1186/s40814-015-0033-z PMC5153673

[58] Green J , Thorogood N . Qualitative Methods for Health Research. Sage; 2018.

[59] Guest G , Namey E , Chen M . A simple method to access and report thematic saturation in qualitative research. PLoS One. 2020;15(5):e0232076. 32369511 10.1371/journal.pone.0232076 PMC7200005

[60] Head E . The ethics and implications of paying participants in qualitative research. Int J Soc Res Methodol. 2009;12:335‐344.

[61] Goodman LA , Liang B , Helms JE , et al. Training counselling psychologists as social justice agents: feminist and multicultural principles in action. Couns Psychol. 2004;32(6):793‐836.

[62] Clarke V , Braun V , Hayfield N . Thematic analysis. In: Smith JA , ed. Qualitative Psychology: A Practical Guide to Research Methods. 3rd ed. Sage Publications; 2015:222‐248.

[63] Guest G . Applied Thematic Analysis. Sage; 2012.

[64] Braun V , Clarke V . To saturate or not to saturate? Questioning data saturation as a useful concept for thematic analysis and sample‐size rationales. Qual Res Sport Exerc Health. 2019;13(2):201‐216. 10.1080/2159676X.2019.1704846

[65] Tookey S , Renzi C , Waller J , von Wagner C , Whitaker KL . Using the candidacy framework to understand how doctor‐patient interactions influence perceived eligibility to seek help for cancer alarm symptoms: a qualitative interview study. BMC Health Serv Res. 2018;18:937. 30514369 10.1186/s12913-018-3730-5 PMC6278141

[66] Greenhalgh T , Wherton J , Papoutsi C , et al. Beyond adoption: a new framework for theorizing and evaluating nonadoption, abandonment, and challenges to the scale‐up, spread and sustainability of health and care technologies. J Med Internet Res. 2017;19:e367. 29092808 10.2196/jmir.8775 PMC5688245

[67] Spencer R , Campbell SM . Tools for primary care patient safety: a narrative review. BMC Fam Pract. 2014;15:166. 10.1186/1471-2296-15-166 25346425 PMC4288623

[68] Abdelrahman W , Abdelmageed A . Medical record keeping: clarity, accuracy, and timeliness are essential. BMJ. 2014;348:f7716. 10.1136/bmj.f7716

[69] Lawton R , O′Hara JK , Sheard L , et al. Can patient involvement improve patient safety? A cluster randomised control trial of the Patient Reporting and Action for a Safe Environment (PRASE) intervention. BMJ Qual Saf. 2017;26:622‐631. 10.1136/bmjqs-2016-005570 PMC5537521 28159854

[70] NHS Improvement . Framework for involving patients in patient safety. 2020. Accessed April 01, 2020. https://engage.improvement.nhs.uk/policy-strategy-and-delivery-management/framework-for-involving-patients-in-patient-safety/user_uploads/200310-draft-framework-for-involving-patients-in-patient-safety.pdf

[71] Morris RL , Kennedy A , Sanders C . Evolving ‘self’‐management: exploring the role of social network typologies on individual long‐term condition management. Health Expect. 2016;19:1044‐1061. 26284341 10.1111/hex.12394 PMC5053258

[72] Osborne RH , Jordan JE , Rogers A . A critical look at the role of self‐management for people with arthritis and other chronic disease. Nat Clin Pract Rheumatol. 2008;4:224‐225. 18301410 10.1038/ncprheum0765

[73] Sanjari M , Bahramnezhad F , Fomani FK , Shoghi M , Cheraghi MA . Ethical challenges of researchers in qualitative studies: the necessity to develop a specific guideline. J Med Ethics Hist Med. 2014;7:14. 25512833 PMC4263394

