# Supplementary material for: Involving patients and carers in patient safety in primary care: A qualitative study of a co‐designed patient safety guide
Source: Health Expect. 2023 Jan 16;26(2):630–9. doi: 10.1111/hex.13673 (PMC10010084; doi:10.1111/hex.13673)
Supplement: Supplementary file 1 — Supporting information. [file HEX-26--s001.docx]

Supplementary material 1

To access all the PSG-PC resources please visit: <https://www.patientsafetyguide.org/>
